# Comprehensive mapping of binding hot spots of SARS-CoV-2 RBD-specific neutralizing antibodies for tracking immune escape variants

**DOI:** 10.1186/s13073-021-00985-w

**Published:** 2021-10-14

**Authors:** Chunyan Yi, Xiaoyu Sun, Yixiao Lin, Chenjian Gu, Longfei Ding, Xiao Lu, Zhuo Yang, Yaguang Zhang, Liyan Ma, Wangpeng Gu, Aidong Qu, Xu Zhou, Xiuling Li, Jianqing Xu, Zhiyang Ling, Youhua Xie, Hongzhou Lu, Bing Sun

**Affiliations:** 1 State Key Laboratory of Cell Biology, Shanghai Institute of Biochemistry and Cell Biology, Center for Excellence in Molecular Cell Science, Chinese Academy of Sciences, Shanghai, 200031 China; 2 Shanghai Public Health Clinical Center, Shanghai Medical College, Fudan University, Shanghai, 201508 China; 3 Key Laboratory of Medical Molecular Virology (MOE/MOH), Department of Medical Microbiology and Parasitology, School of Basic Medical Sciences, Shanghai Institute of Infectious Diseases and Biosecurity, Shanghai Medical College, Fudan University, Shanghai, 200032 China; 4 University of Chinese Academy of Sciences, Beijing, 100049 China; 5 Shanghai Institute of Biological Products Co., Ltd, Shanghai, 200052 China; 6 School of Life Science and Technology, ShanghaiTech University, 100 Haike Road, Shanghai, 201210 China; 7 Bio-Research Innovation Center Suzhou, Shanghai Institute of Biochemistry and Cell Biology, Chinese Academy of Sciences, Suzhou, 215121 China

**Keywords:** SARS-CoV-2, Neutralizing antibodies, RBD antigenic sits, Escape variants

## Abstract

**Background:**

The receptor-binding domain (RBD) variants of SARS-CoV-2 could impair antibody-mediated neutralization of the virus by host immunity; thus, prospective surveillance of antibody escape mutants and understanding the evolution of RBD are urgently needed.

**Methods:**

Using the single B cell cloning technology, we isolated and characterized 93 RBD-specific antibodies from the memory B cells of four COVID-19 convalescent individuals in the early stage of the pandemic. Then, global RBD alanine scanning with a panel of 19 selected neutralizing antibodies (NAbs), including several broadly reactive NAbs, was performed. Furthermore, we assessed the impact of single natural mutation or co-mutations of concern at key positions of RBD on the neutralization escape and ACE2 binding function by recombinant proteins and pseudoviruses.

**Results:**

Thirty-three amino acid positions within four independent antigenic sites (1 to 4) of RBD were identified as valuable indicators of antigenic changes in the RBD. The comprehensive escape mutation map not only confirms the widely circulating strains carrying important immune escape RBD mutations such as K417N, E484K, and L452R, but also facilitates the discovery of new immune escape-enabling mutations such as F486L, N450K, F490S, and R346S. Of note, these escape mutations could not affect the ACE2 binding affinity of RBD, among which L452R even enhanced binding. Furthermore, we showed that RBD co-mutations K417N, E484K, and N501Y present in B.1.351 appear more resistant to NAbs and human convalescent plasma from the early stage of the pandemic, possibly due to an additive effect. Conversely, double mutations E484Q and L452R present in B.1.617.1 variant show partial antibody evasion with no evidence for an additive effect.

**Conclusions:**

Our study provides a global view of the determinants for neutralizing antibody recognition, antigenic conservation, and RBD conformation. The in-depth escape maps may have value for prospective surveillance of SARS-CoV-2 immune escape variants. Special attention should be paid to the accumulation of co-mutations at distinct major antigenic sites. Finally, the new broadly reactive NAbs described here represent new potential opportunities for the prevention and treatment of COVID-19.

**Supplementary Information:**

The online version contains supplementary material available at 10.1186/s13073-021-00985-w.

## Background

Coronavirus disease 2019 (COVID-19), caused by the newly emerging severe acute respiratory syndrome coronavirus-2 (SARS-CoV-2) [1], has spread extensively worldwide [2]. As of September 2021, the global cases of COVID-19 had surpassed 218 million, resulting in more than 4.5 million deaths according to the World Health Organization. During the past year, great achievements have been made in scientific research, especially the development of vaccines and antibody therapies [3, 4]. The receptor-binding domain (RBD) of the spike (S) protein that mediates viral entry by binding with the human cell surface protein angiotensin-converting enzyme 2 (ACE2) is the dominant target of most neutralizing antibodies (NAbs) and vaccines [5, 6].

However, RBD-specific NAbs face a formidable foe. Molecular epidemiology studies have demonstrated that the RBD is highly variable; in particular, the immunodominant receptor-binding motif (RBM) is the most divergent region, and the variations allow the virus to evade the antibody response [7, 8]. Several studies have shown that SARS-CoV-2 had a low genetic barrier to RBD-specific NAb resistance since a variety of independent escape mutations can easily arise in the vesicular stomatitis virus (VSV)-SARS-CoV-2 chimera system under antibody pressure [9, 10]. Therefore, tracking mutations in the RBD region, which could potentially impact COVID-19 progression and treatment strategies, is vital [8, 11].

To better understand the viral emergence and evolution, various viral genomes have been sequenced during the COVID-19 pandemic [12]. Notably, a recent increase in variants with less susceptible to vaccines and increased infectivity caused great concer n[11]. The mutations are featuring RBD. Among the SARS-CoV-2 variants of concern, Beta B.1.351 (RBD-K417N/E484K/N501Y) discovered in South Africa and Gamma P.1 (RBD-K417T/E484K/N501Y) discovered in Brazil have been demonstrated to have high potential to reduce the efficacy of some vaccines [13–15]. Recently, the B.1.617 variant carrying two RBD mutations (E484Q and L452R) emerged in India has become a variant of interest for its high transmission rate and ability to evade immune responses [16]. The RBD mutation on E484 has been associated with resistance to many NAbs [10, 17]. In addition, the global mapping of key residues in the RBD at which amino acid substitutions are associated with immune escape remains unclear. Such information would provide an effective early warning to prevent the rapid and extensive spread of dangerous new SARS-CoV-2 variants. Typically, only a few interacting residues make energetic contributions to antigen-antibody binding [18]; these residues are called “hot spots” [19, 20]. Identification of such hot spot residues recognized by NAbs, especially dominant NAbs, is important for monitoring antibody-based immune escape.

To gain a broad picture of the protective antigenic sites in the RBD, we performed global alanine scanning using several RBD-specific NAbs isolated from COVID-19 convalescents, including several broadly reactive NAbs. Furthermore, we assessed the impacts of natural mutations at key positions on antibody escape and ACE2 binding function. Our study provides a clear experimental reference for monitoring the immune escape of NAbs and vaccines. Additionally, the highly potent NAbs described here represent new opportunities for the prevention and treatment of COVID-19.

## Methods

### Recombinant proteins

To generate recombinant human ACE2-Fc fusion protein (hACE2-Fc), a DNA fragment encoding the extracellular domain of human ACE2 (residues S19 to S740) was fused in-frame with an N-terminal human IgE signal peptide and a C-terminal human IgG1 Fc. There is a flexible “GSGGGG” linker between ACE2 and human IgG1 Fc. The recombinant hACE2 protein was expressed by ExpiCHO-S™ cells (Thermo Fisher Scientific, Cat. No. A2910001) and purified with Protein A (MabSelect Prism A, GE Healthcare) followed by size exclusion chromatography using a Superdex 200 10/300 column (GE Healthcare). To generate recombinant RBD mutants, the DNA sequences encoding the SARS-CoV-2 RBD soluble fragments encompassing amino acids 319-591 of the S protein were fused in-frame with an N-terminal human IgE signal peptide and a C-terminal 8 × His tag. The mutant and wildtype RBD were produced transiently in ExpiCHO-S™ mammalian cells. The proteins were purified by metal affinity chromatography using a His Trap Excel column (GE Healthcare) and dialyzed against phosphate-buffered saline (PBS). The following S proteins expressed either from baculovirus or mammalian expression systems were all utilized for the binding activity assay: SARS-CoV-2 S-ECD-His (Sino Biological, Cat. No. 40589-V08B1) and SARS-CoV RBD-His (Sino Biological, Cat. No. 40150-V08B2).

### Isolation, cloning, expression, and purification of RBD-specific mAbs

This study was approved by the Ethics Review Board of the Shanghai Public Health Clinical Center, Fudan University. Thirty-six COVID-19 convalescent patients and 4 healthy donors were selected randomly in the Shanghai Public Health Clinical Center. The selected COVID-19 patients were aged from 25 to 76 and showed mild symptoms. The blood samples of COVID-19 convalescent patients were collected within 2–3 weeks after discharge. Fasting blood samples were drawn by venepuncture by a medical nurse. We used the blood of COVID-19 convalescent donors for SARS-CoV-2 RBD-specific memory B cell isolation. Human mAbs were generated from human memory B cells by single-cell RT-PCR as previously described [21, 22]. Briefly, peripheral blood mononuclear cells (PBMCs) were stained with Percp-CY5.5-CD4 (BD, Cat. No. 560650), Percp-CY5.5-CD14 (BD, Cat. No. 562692), Percp-CY5.5-CD8 (BD, Cat. No. 565310), FITC-CD19 (BD, Cat. No. 555412), APC-IgG (BD, Cat. No. 550931), and biotinylated RBD-streptavidin-SA BV421 (BD, Cat. No. 563259) before CD19^+^IgG^+^ RBD^+^ single B cells were sorted into 96-well plates containing lysis buffer. The VH, VK, and Vλ variable genes were amplified by RT-PCR and nested PCR using cocktails of specific primers and then cloned into expression vectors [22]. The gene sequence analysis of mAbs was performed by IMGT and IgBLAST. The S309, CR3022, CB6, and B38 VH and VL sequences were synthesized and cloned into expression vectors (Shanghai Generay Biotech Co., Ltd). The plasmids encoding the paired heavy chain and light chain were cotransfected into ExpiCHO™ cells according to the manufacturer’s instructions. After 7 days, the antibodies were purified from the ExpiCHO™ cell supernatants using Protein A.

### ELISA

To determine the binding activities of the antibodies or plasma, recombinant protein (0.5 μg/ml in 100 μl/well) was captured in a 96-well plate overnight, and the plate was blocked with 2% bovine serum albumin (BSA) in PBS-Tween 20 (PBST) for 2 h. The antibodies or plasma samples were serially diluted in PBST and incubated in the wells for 2 h. The samples were washed three times, and an anti-human IgG1 Fc HRP antibody (Sigma-Aldrich) was used to detect the binding affinity. The absorbance at 450 nm was recorded by a plate reader (Bio-tek). To assess the reactivity of SARS-CoV-2 RBD-specific antibodies to the denatured RBD, ELISA was performed as mentioned above. The RBD was denatured with 0.1% SDS, 50 mM DTT, and a metal water bath at 100°C for 5 min. Anti-HCV 8D6 IgG1 was used as an isotype control [21].

For the receptor-blocking assay, hACE2-Fc (5 μg/ml) was coated onto microplates. The isolated antibodies or plasma samples were serially diluted and incubated with the biotinylated RBD (200 ng/ml) for 1 h and then added to the wells after washing and blocking. HRP-conjugated streptavidin (R&D Systems, Cat. No. DY998) was used as the detection antibody. The following procedure was the same as mentioned above. The percentage of binding inhibition was calculated as the percent reduction in RBD binding to hACE2-Fc from the value in the absence of the antibody.

For antibody competition assays, excessive amounts of primary antibodies (50 μg/ml) were added to the recombinant RBD pre-coated plates at a concentration of 0.5 μg/ml, and the plates were incubated for 1 h at 37°C. Biotin-labeled secondary detection mAbs (1 μg/ml) were then added to the plates. The plates were washed after 2 h of incubation at 37°C, and binding of the detection antibodies was detected with HRP-conjugated streptavidin.

### RBD mutagenesis and binding measurements

The DNA sequences encoding the SARS-CoV-2 RBD soluble fragments encompassing amino acids 319-589 of the S protein were fused in-frame with an N-terminal human IgE signal peptide and a C-terminal human IgG1 Fc and 8 × His tag. Global alanine scanning of the RBD was performed using site-directed mutagenesis of RBD residues (330-521) to alanine (natural mutations for alanine residues). Substitutions of the residues at the antigenic sites selected for mutagenesis were based on the 2019 Novel Coronavirus Resource (2019nCoVR) released by the China National Center for Bioinformation (https://ngdc.cncb.ac.cn/ncov/variation/spike). Site-directed mutagenesis was induced with a commercialized KOD-Plus mutagenesis kit (TOYOBO, Cat. No. SMK-101). All of the mutations were confirmed by DNA sequence analysis (Biosune). The resulting plasmids were transfected into ExpiCHO-S™ cells in 12-well plates. The supernatants were harvested 96 h after transfection. Sandwich ELISA was performed to measure individual RBD protein expression in the cell supernatant. In brief, a mouse anti-6 × His tag mAb (proteintech, Cat. No. 66005-1-Ig) was used to coat plates; 20-, 100-, and 500-fold dilutions of cell supernatant were added. Serially diluted purified RBD-Fc-8× His (2-fold dilutions from 500 ng/ml) was used as a standard, and detection was performed with HRP-conjugated goat anti-human IgG1 Fc (Sigma, Cat. No. A0170). The concentration of the sample was calculated according to a standard curve generated from the serial dilution data. Another ELISA was performed to analyze the relative binding activities of these RBD mutants for RBD-specific antibodies and ACE2; wildtype RBD was used as a control. Then, 300 ng/ml concentrations of the RBD mutants were incubated with plates pre-coated with 500 ng of antibodies or 500 ng of ACE2-Fc, and detection was performed with an HRP-conjugated mouse anti-6× His mAb (proteintech, Cat. No. HRP-66005). The signals produced by binding of the mutants to the mAbs were compared to those produced by binding of the wildtype.

### Biolayer interferometry (BLI) analysis of RBD and antibody binding affinity

Binding affinity (KD) analysis was conducted by BLI at 25°C using an Octet Red 96 system (ForteBio, Inc.) as previously described [21]. mAbs (20 μg/ml) were captured on an anti-human IgG-Fc (AHC)-coated biosensor surface for 5 min. The baseline interference was then read for 60 s in kinetics buffer (KB: 1× PBS, 0.01% BSA, and 0.02% Tween-20), after which the sensors were immersed into wells containing recombinant RBD diluted in KB for 300 s (association phase). The sensors were then immersed in KB for the indicated times (dissociation phase) for up to 900 s. The mean *K*_on_, *K*_off_, and apparent KD values were calculated using an equation globally fitted to a 1:1 binding kinetic model with an *R*^2^ value of ≥0.95.

### Surface plasmon resonance (SPR) analysis of RBD mutants and ACE2-Fc

The affinity of RBD mutants for ACE2-Fc was measured using SPR. Purified ACE2-Fc was captured on a Protein A sensor chip using a BIAcore 8K chip (GE Healthcare). RBD samples at the following concentrations were prepared and injected at 100 μl/min: 0 nM, 6.25 nM, 12.5 nM, 25 nM, 50 nM, 100 nM, and 200 nM. The BIAcore chip was regenerated between each cycle with a regeneration buffer containing 10 mM glycine (pH 1.5). The generated binding curves were used to extract the kinetics of the rate of RBD binding to ACE2-Fc. Due to the slow off-rate of this interaction, separate extended dissociation off-rate experiments were performed. The data were double reference-subtracted and fit using a 1:1 binding model.

### Pseudovirus neutralization assay

For pseudovirus variants, individual mutations associated with RBD antibody escape were introduced into a full-length SARS-CoV S plasmid using homologous recombination. All of the mutations were confirmed by DNA sequence analysis (Biosune). SARS-CoV, SARS-CoV-2, and SARS-CoV-2 variants of pseudovirus were produced as previously described [23]. Briefly, plasmids encoding the full-length S gene and pNL4-3.luc.RE were cotransfected into HEK 293T cells in 10-cm dishes with Lipofectamine 3000 Transfection Reagent. The medium was replaced after 6 h, and the virus supernatants were collected 48 h after transfection. HEK 293T-hACE2 cells were plated into 96-well plates 1 day prior. The constructed pseudovirus was quantified and normalized by HIV-1 p24 ELISA (Biodragon, Cat. No. BF06203). After adjustment, equal titers of viruses (equivalent to 20 ng/ml of p24 Ag) were then diluted into in DMEM with 10% FBS, mixed with an equal volume of serially diluted antibodies or plasma (preheated at 56°C for 30 min to inactivate complement), and incubated for 1 h at 37°C. The mixtures were transferred to HEK 293T-hACE2 cells. The cells were incubated at 37°C for 48 h and then subjected to cell lysis and a luciferase activity assay (Promega). The percent neutralization was calculated by comparing the luciferase value of the antibody-containing samples to those of the untreated controls. The neutralization curves were fit by nonlinear regression using GraphPad Prism software.

### Authentic virus neutralization assay

An authentic virus neutralization assay was performed in the BSL-3 laboratory of Fudan University in compliance with the guidelines of the laboratory biosafety manual. The SARS-CoV-2 clinical isolate nCoV-SH01 (GenBank: MT121215.1) was amplified in Vero E6 cells and used for authentic virus neutralization [24]. Live SARS-CoV-2 virus (100 TCID50, 50 μl) was mixed with 50 μl of threefold serially diluted RBD-specific NAbs and incubated at 37°C for 1 h. The supernatant was discarded, and the antibody-virus mixture was transferred into Vero E6 cells. After incubation at 37°C for 1 h, 100 μl of the mixture at each dilution was added in duplicate to Vero E6 cells in a 96-well plate. After incubation for 2 days at 37°C in a humidified atmosphere with 5% CO2, the plates were inspected under an inverted optical microscope. The highest plasma dilution that protected more than 50% of cells from cytopathic effect (CPE) was taken as the neutralization titer. The neutralization curves were fit by nonlinear regression using GraphPad Prism software.

### Structure analysis

Structural figures were generated using the PyMOL Molecular Graphics System (Version 2.0, Schrödinger, LLC). The programs MAESTRO [25] and DUET [26] were used to predict the stability of RBD alanine mutants based on the RBD structure (Protein Data Bank [PDB] ID code 7C01).

### Bioinformatic sequencing analysis

The sequences were aligned using MUSCLE in MEGA7 [27] or ClustalX [28], and the percent conservation for each residue is presented as the quality score of each column. The time-frequency data of RBD mutants were generated and provided by the National Genomics Data Center (NGDC) [29, 30]. The RBD mutations present in evaluated SARS-CoV-2 genomic sequences deposited in GISAID and GenBank as of June 18, 2020, were analyzed by CoVsurver [31, 32].

## Results

### Isolation and characterization of RBD-specific NAbs from COVID-19 convalescent donors

**Fig. 1:**
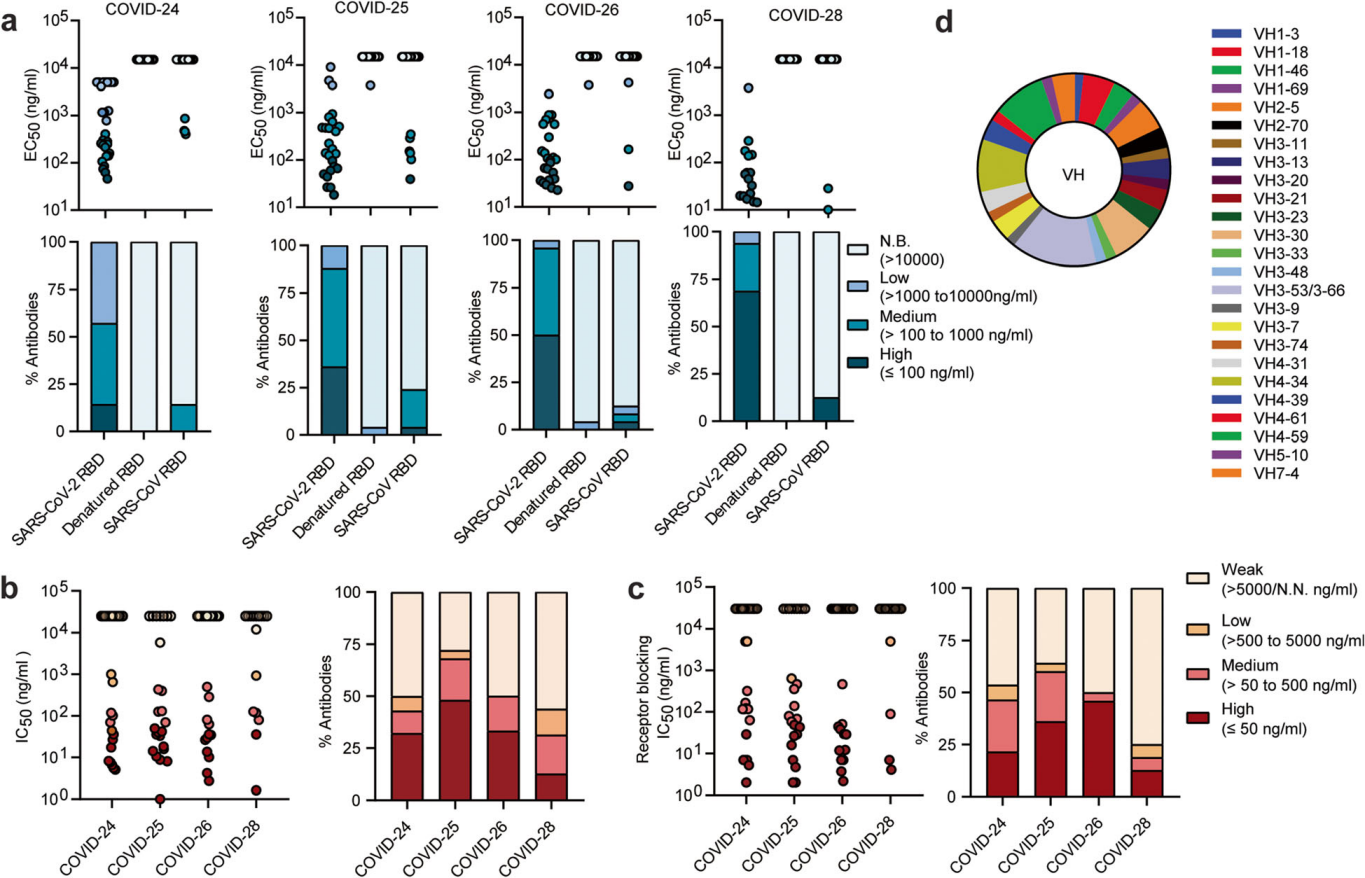
Characterization of SARS-CoV-2 natural infection-induced RBD-specific mAbs. **a** Antibody binding activity (*EC*_*50*_) with native and denatured SARS-CoV-2 RBD as well as native RBD of SARS-CoV was measured by ELISA. Upper panel, four ranges per donor; lower panel, percentage of mAbs from each donor with the indicated *EC*_*50*_ range. N.B., non-binding activity. **b** SARS-CoV-2 pseudovirus neutralization potency (*IC*_*5*0_) (left). Percentage of antibodies with indicated neutralization potencies (right). Results are derived from a single experiment performed in triplicate. **c** ACE2 blocking activity (*IC*_*50*_) (left). Percentage of antibodies with indicated receptor-blocking potencies (right). For **b** and **c**, N.N., non-neutralization or non-blocking activity. The data represent one representative experiment of two independent experiments. **d** Distribution of heavy-chain variable (VH) germline genes of RBD-specific NAbs

To elucidate the degree to which SARS-CoV-2 will adapt to evade NAbs, we first generated RBD-specific NAbs by screening a cohort of 36 convalescent patients infected with early-circulating SARS-CoV-2 strains during January–March 2020 and selected four donors with high titers of plasma RBD-binding antibodies and NAbs against a SARS-CoV-2 pseudovirus (Additional file 1: Fig. S1). We then conducted single-cell PCR experiments to generate human monoclonal antibodies (mAbs) from memory B cells using the SARS-CoV-2 RBD as the bait. Ninety-three RBD-specific antibodies were identified (Fig. 1a and Additional file 2: Table S1). More than 95% of the RBD-specific mAbs did not bind to the denatured form of RBD, indicating that the epitopes targeted by RBD-specific antibodies induced by natural infection are highly dependent on conformation. Furthermore, approximately 12–24% of isolated mAbs from a given donor showed cross-reactivity with SARS-CoV; these findings are in line with previous studies suggesting that cross-reactive mAbs could be induced by natural infection [33–35], as the SARS-CoV-2 and SARS-CoV RBDs share 76% amino acid identity [23].

Remarkably, 50–72% of the mAbs from each donor exhibited detectable pseudovirus SARS-CoV-2 neutralization (*IC*_*50*_ < 5 μg/ml), with 12–48% designated high-potency mAbs (*IC*_*50*_ ≤ 50 ng/ml) (Fig. 1b and Additional file 2: Table S1). Expectedly, 25–64% of the mAbs from each donor had measurable receptor-blocking activity (*IC*_*50*_ < 5 μg/ml), suggesting that most of the RBD-specific NAbs protect against virus infection via mechanisms that block attachment to the cellular receptor ACE2 (Fig. 1c). More importantly, three antibodies (28-26K, 25-F7, and 25-D9) potently neutralized the SARS-CoV-2 pseudovirus with an *IC*_*50*_ of 14–25 ng/ml and moderately neutralized the SARS-CoV pseudovirus with an I*C*_*50*_ of 0.9–5 μg/ml. They efficiently blocked binding of the SARS-CoV-2 and SARS-CoV RBDs to ACE2 (Additional file 1: Fig. S2 and Table 1), unlike a previously described broadly reactive NAb, S309, which was independent of receptor-binding inhibition [36]. Like a known cross-protective antibody COVA1-16, these rare cross-neutralizing antibodies bind to the conserved non-RBM epitope [37].

**Table 1:** Binding affinity and neutralization potency characterization of the selected NAbs

Antigen specific	mAb	SARS-CoV-2				SARS-CoV		
		RBD KD (nM)	Receptor blocking *IC*_*50*_ (ng/ml)	Pesudo virus *IC*_*50*_ (ng/ml)	Authentic virus *IC*_*50*_ (ng/ml)	RBD KD (nM)	Receptor blocking *IC*_*50*_ (μg/ml)	Pesudo virus *IC*_*50*_ (μg/ml)
**SARS-CoV-2 specific**	24-2K	6.1	63.4	17.0	171.0	N.D.	N.D.	N.D.
	24-1L	7.3	7.0	5.1	229.0	N.D.	N.D.	N.D.
	24-11K	2.7	2.0	5.6	74.0	N.D.	N.D.	N.D.
	24-12K	2.2	7.0	8.2	162.0	N.D.	N.D.	N.D.
	25-C4	1.2	43.2	50.0	112.0	N.D.	N.D.	N.D.
	25-C5	1.0	49.0	33.0	419.0	N.D.	N.D.	N.D.
	25-C8	2.8	68.6	68.0	345.0	N.D.	N.D.	N.D.
	25-C9	2.2	138.9	8.0	146.0	N.D.	N.D.	N.D.
	25-B5	3.5	2.0	1.0	91.0	N.D.	N.D.	N.D.
	25-F8	4.0	79.9	8.7	345.0	N.D.	N.D.	N.D.
	25-G7	0.5	29.0	10.6	55.0	N.D.	N.D.	N.D.
	26-40K	6.8	2.2	4.2	21.0	N.D.	N.D.	N.D.
	26-34L	15.9	7.0	10.0	171.0	N.D.	N.D.	N.D.
	26-45K	3.9	3.7	2.7	162.0	N.D.	N.D.	N.D.
	28-15L	2.7	7.0	35.0	171.0	N.D.	N.D.	N.D.
	28-8L	11.9	4.1	4.0	38.0	N.D.	N.D.	N.D.
	CB6	10.7	7.0	9.7	73.0	N.D.	N.D.	N.D.
**Crossing**	25-D9	0.4	356.1	14.0	345.0	7.2	2.2	0.9
	25-F7	1.2	26.5	22.0	1883.0	4.9	5.3	5.0
	28-26K	0.1	89.3	25.0	330.0	4.9	3.8	3.5
	CR3022	57.8	>15000	>25000	N.D.	0.9	1.9	0.4
	S309	<1E-3	>15000	60.2	N.D.	<1E−3	>15	0.03

*N.D.*, not determined; CB6, CR3022, and S309 were used as control [36, 38, 39]

The NAbs were nearly unrestricted in the germline gene repertoire. Among these heavy chains, 73% originated from IGHV3 and IGHV4 (Fig. 1d and Additional file 3: Table S2), which are also the highest frequency in the antibody repertoire of healthy donors [40]. We also observed that RBD-binding NAbs were strongly biased towards IGHV3-53/3-66, consistent with the findings of previous studies [4, 41, 42], which suggests that they play an important role in the humoral immune response to SARS-CoV-2 infection [43].

Finally, we verified that the 19 selected mAbs could efficiently neutralize authentic SARS-CoV-2 infection with *IC*_*50*_ values from 20 ng/ml to 1.8 μg/ml, and we found that several NAbs exhibited neutralizing activity comparable to that of CB6, which is in clinical [38]. The 19 NAbs were used as probes to search for the binding determinants of RBD-specific NAbs due to their high affinity for the SARS-CoV-2 RBD and potent neutralization of SARS-CoV-2 (Table 1 and Additional file 1: Fig. S3).

### Mapping and characterization of protective antigenic sites and antibody binding hot spots on RBD

To define the epitopes recognized by selected RBD-specific NAbs, we first performed competitive binding experiments. Three well-described mAbs targeting independent epitopes, CB6, CR3022 and S309, were used as controls [36, 38, 39]. Our panel of NAbs could be classified into 5 groups (Table 2). The group 1 and group 2 antibodies competed with CB6, while the group 4 antibodies competed with S309. The three cross-reactive antibodies in group 5 competed with CR3022. The antibodies in group 2 and group 3 may have larger footprints than those in the other groups because they competed with antibodies from two of the other groups. Interestingly, 4/5 of the group 1 antibodies utilized VH3-53/3-66 and had short CDRH3 lengths of 9–13 amino acids (Additional file 1: Table S3).

**Table 2:**
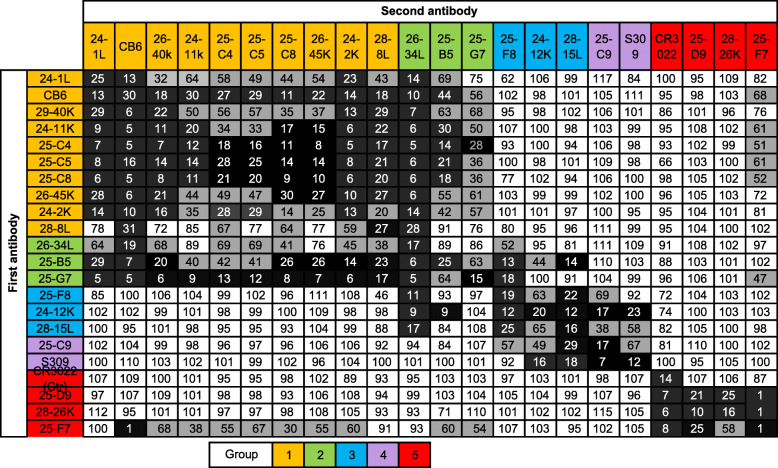
Epitope binning for the selected NAbs by competitive ELISA

Data indicate the percent binding of the second antibody in the presence of the blocking antibody, as compared to that of the second antibody alone. Cells filled in black indicate full competition, in which ≤ 30% of the uncompeted signal is observed; gray indicates intermediate competition, if the signal is between 30 and 70%; and white indicates non-competing, if the signal is ≥ 70%. Antibodies are classified into five groups in different colors based on competition-binding with the control mAbs CB6 (group 1), S309 (group 4), or CR3022 (group 5). Antibodies in group 2 compete with antibodies in group 1 and group 3, while antibodies in group 3 compete with antibodies in group 2 and group 4

**Fig. 2:**
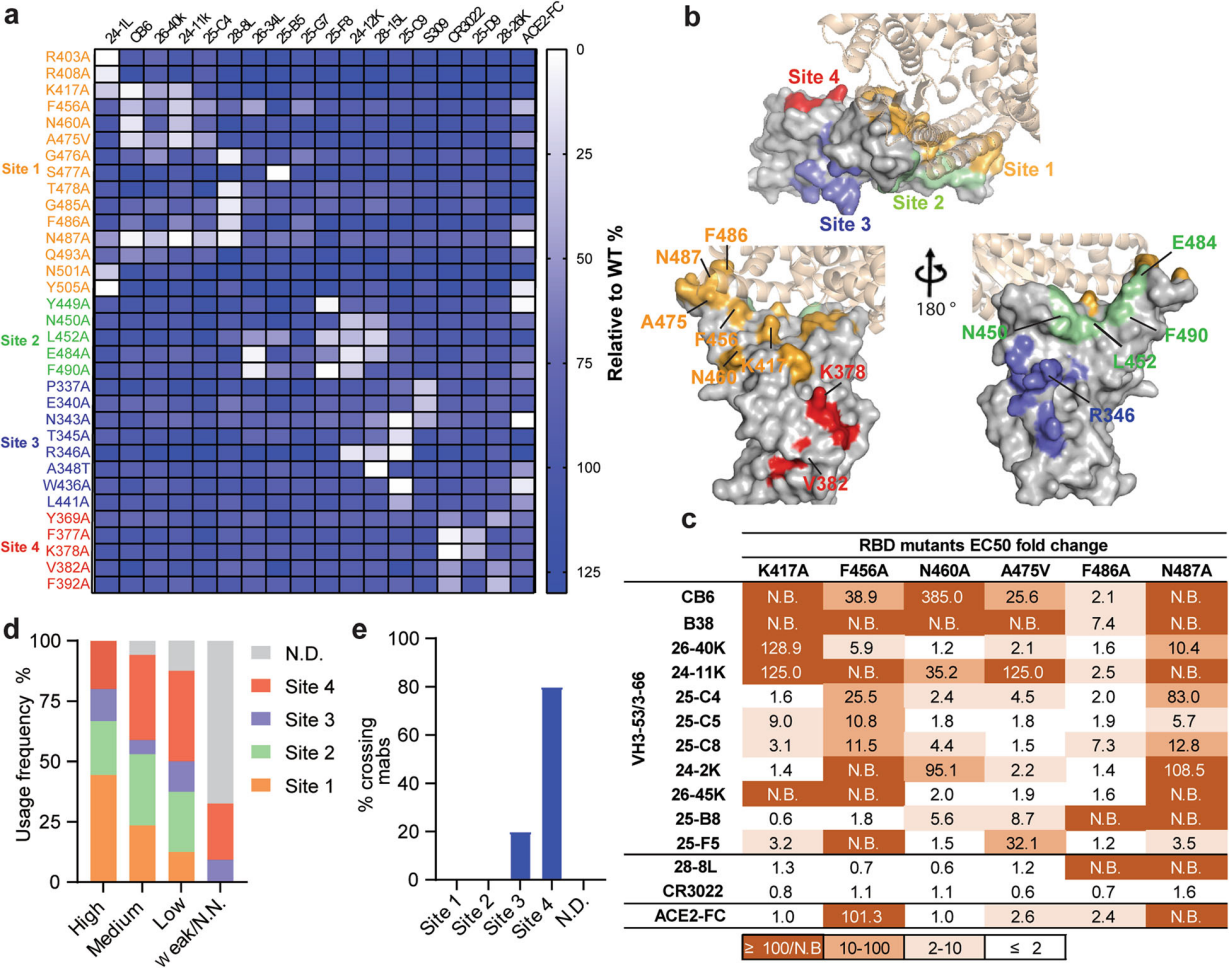
Determination and characterization of the antigenic sites on RBD. **a** Mapping of binding sites of a panel of RBD-specific NAbs by global alanine scanning. Thirty-three amino acid positions were identified in four antigenic sites (1–4) on RBD as main targets for RBD-specific NAbs. Degree of binding reduction was defined as percentage by OD_450_ of each mutant relative to OD_450_ of RBD wildtype and is represented as a heatmap from white (reduction) to blue (no impact). The data are representative of at least two independent experiments. **b** Location of four distinct antigenic sites on the RBD region (PDB ID: 6M0J). The color-coding scheme is described as follows: site 1 (orange), site 2 (green), site 3 (slate blue), site 4 (red); ACE2 (wheat). Top and down are shown from different angles. The key hot spots targeted by NAbs are shown. **c** Key residues for VH3-53/3-66 dominant NAb recognition. Binding fold change was calculated as follows: *EC*_*50*_ of RBD mutant/*EC*_*50*_ of RBD wildtype. **d** Antibodies are grouped according to neutralization potency and colored by the usage frequency of each antigenic site. Each antibody was tested for competition with a panel of known antibodies and assigned to an antigenic site based on the competition profile. **e** Percentage of SARS-CoV-2 and SARS-CoV crossing reactive antibodies targeting each antigenic site. The data represent one representative experiment of two independent experiments

To obtain a comprehensive view of the antigenic sites on the SARS-CoV-2 RBD and the determinants of RBD NAb recognition, we performed global RBD alanine scanning mutagenesis (at nearly 190 RBD amino acid positions) with a panel of 17 mAbs derived from the five groups. Functional epitope mapping identified 33 binding determinants for NAbs and defined four major antigenic sites (1–4) targeted by RBD-specific NAbs based on their structural locations and epitope competition results. Site 1 and site 2 overlap with ACE2 binding sites, while site 3 and site 4 are located outside the sites (Fig. 2a, b and Additional file 1: Table S4). Some antibodies bind to only one site, whereas others contact more.

The group 1 antibodies mainly bound to site 1. We demonstrated that some mutations, namely, K417A, F456A, N460A, A475V, F486A, and N487A, led to less binding for multiple group 1 antibodies. To verify whether these residues were crucial for the dominant VH3-53/3-66-encoded antibodies, we selected eleven VH3-53/3-66-encoded antibodies, including CB6 and B38 antibodies whose structures have previously been characterized [38, 44], as probes (Additional file 1: Fig. S4a). We demonstrated that most of the VH3-53/3-66-encoded NAb prototypes were sensitive to mutations at F456 and N487 in different extents, while K417, N460, A475, and F486 were also involved in the contact of some VH3-53/3-66-encoded NAbs (Fig. 2c). The heavy chains of CB6 and B38 use a similar structural mode for epitope recognition (Additional file 1: Fig. S4b). The conserved germline-encoded CDRH1 and CDRH2 together with the distinct CDRH3 contribute to tight contact with the core epitope on the RBD formed by K417, F456, N460, A475, and N487 residues within antigenic site 1, suggesting that mutations at these positions may give rise to resistance to VH3-53/3-66-prototype NAbs. With regard to the site 2 binding region, we observed that substitutions at shared positions (N450, L452, E484, and F490) reduced the binding of NAbs in groups 2 and 3 to the RBD. In addition, R346 within antigenic site 3 showed favorable interactions with three mAbs from group 3 and group 4. Antibodies in group 5 distinctly preferentially bound to the residues within site 4 (Fig. 2a). We further investigated whether the identified residues involved in antibody binding also influence ACE2 binding (Fig. 2a). As expected, substantial loss of ACE2 binding was caused by mutations in surface residues, namely, F456, F486, N487, Y505, and Y449, each of which is involved in ACE2 binding according to structural analysis [5, 45]. Two core region mutations distant from the ACE2 binding surface, N343A and W436A, also resulted in the loss of ACE2 binding.

We analyzed the relationship between neutralization potency and each antigenic site (Fig. 2d and Additional file 4: Table S5). Over 70% of the highly potent NAbs targeted antigenic sites 1 and 2; thus, antigenic sites 1 and 2 are the prime targets of SARS-CoV-2 neutralizing antibodies. Furthermore, SARS-CoV-2 and SARS-CoV cross-reactive mAbs mainly targeted antigenic sites 3 and 4 (Fig. 2e), indicating that these two sites are conserved exposed sites, consistent with the findings of previous studies [36, 39]. Taken together, the results have implications for the design of SARS-CoV-2 vaccines, and the binding hot spots of RBD-specific NAbs identified here will support direct and intentional monitoring of immune escape mutants.

### The residues essential for RBD folding and antigen conformation are evolutionarily conserved among sarbecoviruses

**Fig. 3:**
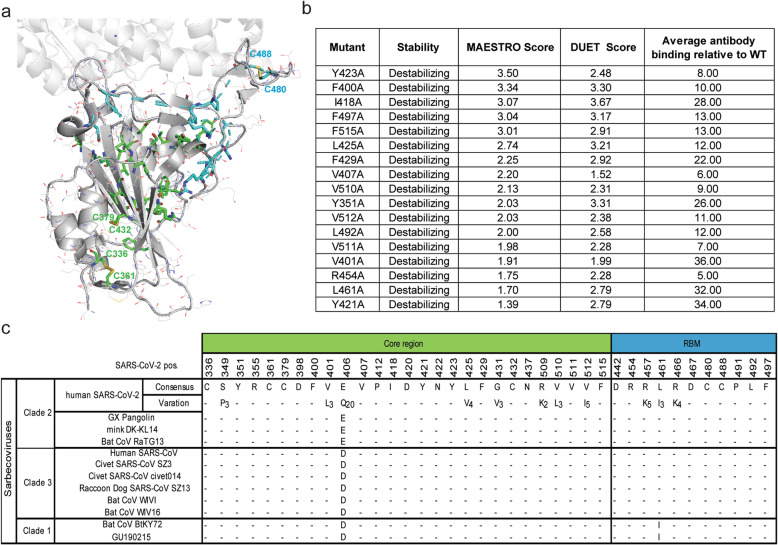
The mutations that impair the global folding of RBD are evolutionally conserved. **a** The residues of which alanine substitution impaired RBD antigenic conformation and ACE2 binding ability are shown in a surface representation (PDB ID: 6M0J). Mutants located in the core region are highlighted in green and RBM are in cyan. Yellow sticks indicate disulfide bridges. **b** Conservation of residues that are essential for RBD folding across clades of sarbecoviruses. Human and animal SARS-related coronaviruses were classified by clades. SARS-CoV-2 genome sequences (*n* = 364,409) retrieved from GISAID and Genbank on January 19, 2021 (*n* = 11,839) and human SARS-CoV genome sequences (*n* = 200) from Genbank were used to annotate variants of the spike glycoprotein. Dashes indicate identity to SARS-CoV-2 consensus residues. Variants found in at least two sequences are parenthesized. **c** Top predicated destabilizing mutants by structural analysis. The score of MAESTRO and DUET is for predicted impact on stability from the RBD structure (PDB ID: 7C01). Average percent binding versus wildtype RBD for conformation-dependent RBD antibodies are shown

Our landscape of mapping data also demonstrated that mutations at approximately 20% of the positions (38 of 190 positions) led to substantial loss of binding for nearly all NAbs as well as recombinant hACE2 (Additional file 1: Table S6). Most of these residues were buried within the RBD core structure, thus were likely to facilitate RBD folding (Fig. 3a). Recent studies indicated that global RBD stability contributes to ACE2-binding affinity [46], and our data further revealed that global RBD stability is also essential for the binding of some antibodies. Furthermore, these residues are highly conserved across clade 1, 2, and 3 sarbecoviruses [47, 48], including human and animal isolates (Fig. 3b). Additionally, point mutations can strongly affect protein stability, which may in turn affect protein function, as illustrated by studies on other viruses [20, 49]. The top 17 destabilizing mutations predicted by the two structure-based methods MAESTRO [25] and DUET [26] showed high free energy change (ΔΔG) values and low average antibody binding percentages. Mutations resulting in improper RBD folding should be considered in determining the functional epitope of antibodies by alanine scanning. Based on structural analysis of the RBD-CB6 complex and the RBD-B38 complex, the conserved Y421 residue is part of the epitope of these complexes because it forms hydrogen bonds with G54 in the CDRH2 of CB6 and B38 (Additional file 1: Fig. S4b). Collectively, the data suggest that some residues may have low mutational tolerance, so targeting these positions with antibodies could limit viral escape.

### Natural substitutions of antibody binding hot spots confer resistance to multiple NAbs

To investigate the residue polymorphism of each antigenic site, we computed the sequence variability at positions that were binding determinants for selected mAbs (Additional file 1: Fig. S5). The data showed that site 1 and site 2 were more polymorphic than site 3 and site 4. Mutations were more frequently introduced in the positions with variable sequences between SARS-CoV-2 and SARS-CoV such that multiple sites were replaced by the same residues or by residues with similar biophysical properties at the corresponding positions of SARS-CoV RBD. Additionally, residues at positions 417, 475, 484, 452, 490, and 346, which are key recognition sites for multiple NAbs, were highly polymorphic. In contrast, some conserved residues that were proven to be critical for ACE2 binding by alanine scanning, such as N487, Y505, Y449, W436, and N343 (Fig. 2a), had limited variability, suggesting that these residues have a low inherent tolerance for mutations.

**Fig. 4:**
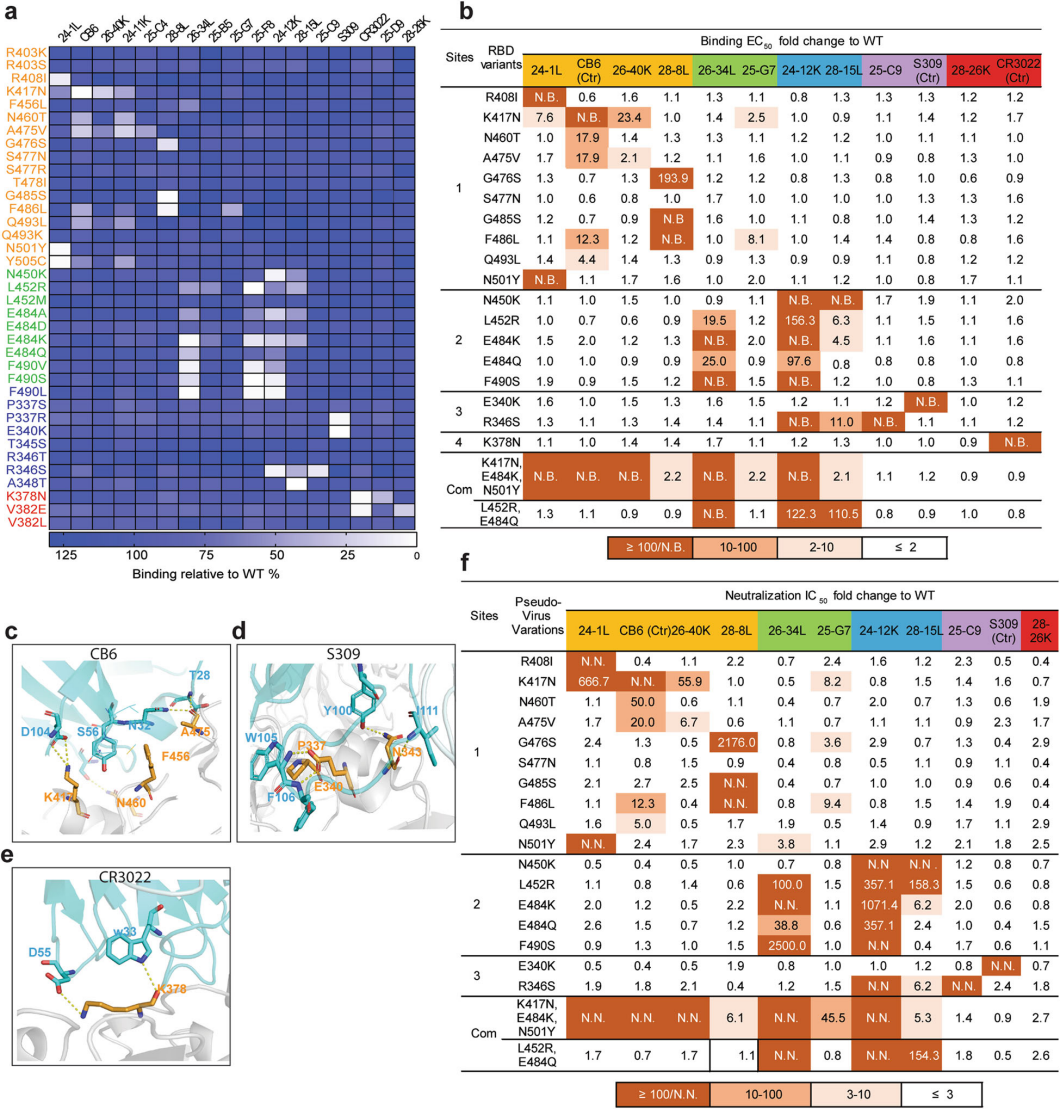
The impacts of natural mutations at antigenic sites on binding and neutralizing activities of RBD-specific NAbs. **a** Binding activity of RBD natural mutants with a panel of NAbs was evaluated by ELISA. Degree of binding reduction was defined as percentage by OD_450_ of each mutant relative to OD_450_ of RBD wildtype is represented as a heatmap from white (reduction) to blue (no impact). The data are representative of at least two independent experiments. **b** Binding of 18 purified mammalian expressed RBD single-point mutants and two co-mutations with a panel of NAbs. Binding fold change was calculated as follows: *EC*_*50*_ of RBD mutant/*EC*_*50*_ of RBD wildtype. N.B., not binding. **c** Details of the structural interaction between CB6 and RBD (PDB ID: 7C01). **d** Details of the structural interaction between S309 and RBD (PDB ID: 6WPT). **e** Details of the structural interaction between CR3022 and RBD (PDB ID:7A5S). For **c**–**e**, polar interactions are indicated by yellow dashed lines. Cyan, heavy chain; light blue, light chain; gray, RBD. Key residues on RBD highlighted in orange sticks; key residues on heavy chain highlighted in cyan sticks. **f** Neutralization of 19 pseudotyped variants by a panel of RBD-specific NAbs. Neutralization fold change was calculated as follows: *IC*_*50*_ of pseudotyped variants/*IC*_*50*_ of the wildtype. *IC*_*50*_ values were calculated from three independent experiments. The data represent one representative experiment of two independent experiments

To explore the impacts of natural mutations on NAb binding, we constructed and expressed RBDs with single-amino acid substitutions that are present in circulating human isolates of SARS-CoV-2. For residues at which several alternate amino acids with different side chains were selected, the different substitutions did not contribute equally to NAb binding (Fig. 4a and Additional file 1: Table S7). For example, alanine scanning revealed that the F456A mutation caused loss of binding of VH3-53/3-66 NAbs, but the natural F456L variation did not result in resistance to VH3-53/3-66 NAbs. We also observed that L452R rather than L452M led to substantial loss of binding to 24-34L, 25-F8, 24-12K, and 28-15L. E484A and E484K resulted in resistance to 26-34L, 24-12K, and 25-F8 while E484Q showed a mild impact and E484D had no impact. F490V, F490S, and F490L each resulted in strong resistance to 26-34L, 25-F8, and 24-12K. P337R instead of P337S conferred resistance to S309, and R346S instead of R346T caused a significant loss of binding of 24-12K, 28-15L, and 25-C9. V382E rather than V382L reduced the binding activity of 28-26K and CR3022 in group 5. Collectively, the data suggest that different properties of amino acid substitutions, including hydrophobicity, polarity, and charge, might determine resistance in terms of requirements for interactions with mAbs.

We further purified 18 RBD single-point mutants and 2 co-mutations expressed by mammalian cells and measured their binding activity with the panel of NAbs (Fig. 4b). The fold changes in the *EC*_*50*_ values compared to those of the RBD wildtype were investigated, and the results were consistent with those of our preliminary mutational scan. Moreover, the molecular mechanisms of the effects of the mutations on three well-characterized mAbs, CB6, S309, and CR3022, were well explained by the structures (Fig. 4c–e). For example, the replacement of K417 with Asn (N) greatly weakened CB6 binding affinity by disrupting a strong salt bridge between K417 in the SARS-CoV-2 RBD and the CB6 CDRH3. However, E340K and K378N disrupted the key hydrogen bonds with S309 and CR3022, respectively.

To further investigate whether the binding escape mutants exhibited NAb resistance, we constructed a panel of 19 SARS-CoV-2 pseudovirus variants, including 17 single RBD mutants and 2 co-mutations, to examine their impacts on the neutralization potency of the 12 NAbs mentioned above (Fig. 4f). Since the dominant S sequence variant seen in clinical isolates is D614G, all the SARS-CoV-2 pseudovirus variants we constructed were coupled with the D614G variant [50]. As expected, in agreement with the *EC*_*50*_ value results (in which the RBD substitutions resulted in high *EC*_*50*_ values for NAbs), the pseudovirus variants correspondingly conferred resistance to NAbs with high *IC*_*50*_ values. Notably, the most frequent RBD variants seen in clinical isolates, N501Y and S477N, remained similarly sensitive to the majority of the selected NAbs; only 24-1L failed to neutralize N501Y. Substitutions responsible for major antigenic escape were in antigenic site 1 (K417N, F486L), antigenic site 2 (N450K, E484K, E484Q, L452R, F490S), and antigenic site 3 (R346S). Using our immune escape mapping strategy, we identified a natural mutant, E340K, in the circulating virus that conferred resistance to a broadly reactive NAb, S309, and five mutants that resulted in resistance to CB6. These findings could inform the therapeutic use of these antibodies in clinical studies. Expectedly, the B.1.351 variant (RBD-K417N/E484K/N501Y) facilitated resistance to a somewhat wider range of NAbs than single mutations, which conferred complete resistance to five highly potent NAbs targeting major antigenic sites (site 1 and site 2) in terms of binding and neutralizing activity. However, the site 3- and site 4-targeting antibodies retained their ability to neutralize the B.1.351 pseudovirus. On the contrary, the RBD double mutants E484Q and L452R within antigenic 2 which featured by B.1.617.1 [16] do not cause substantial antibody evasion.

### Impact of RBD mutations on ACE2 binding affinity

**Fig. 5:**
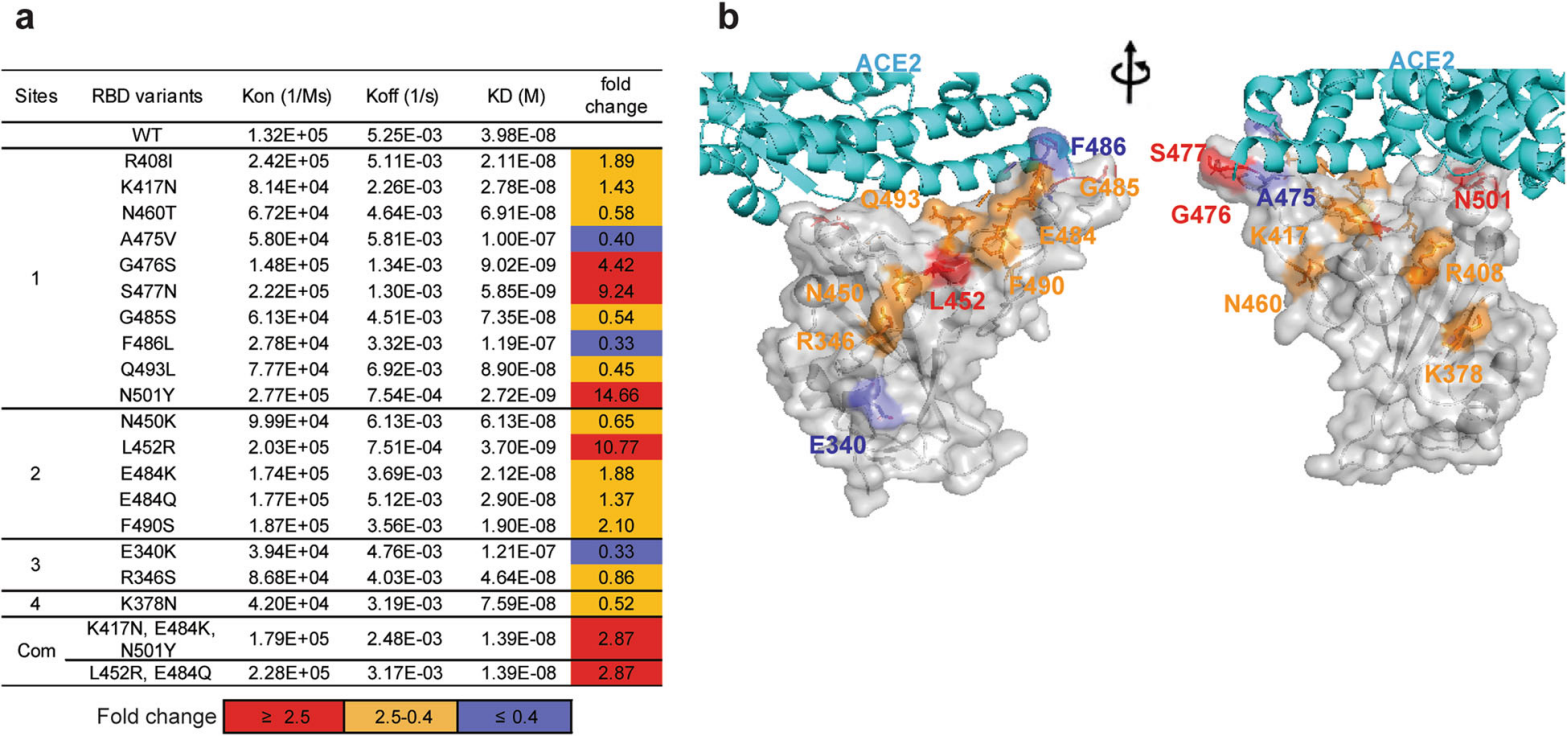
The binding affinity between antibody escape mutants and ACE2. **a** Affinity measurement of purified RBD mutants for binding to immobilized ACE2-Fc by surface plasmon resonance (SPR). The *K*_on_ and *K*_off_ were determined by BIAcore and the KD were computed as *K*_off_/*K*_on_. Neutralization fold change was calculated as follows: KD value of RBD wildtype/KD value of RBD mutants. **b** The residues that are important for resistance to antibodies are presented on the interface of the ACE2 and RBD. The position of mutants that enhance ACE2 binding affinity are highlighted in red (affinity fold change ≥ 2.5); comparable to wildtype are in orange (affinity fold change between 2.5 and 0.4); reduce ACE2 binding affinity are in slate blue (affinity fold change ≤ 0.4). The data represent one representative experiment of two independent experiments

To investigate how the antigenic escape residues identified by our study affect the RBD-ACE2 interaction, the binding affinities of eighteen mutated RBDs to ACE2 were analysed with BIAcore 8K (Fig. 5a and Additional file 1: Fig. S6). The majority of the mutants retained or even exhibited enhanced hACE2 binding. N501Y, L452R, and S477N mutants exhibited high affinity for ACE2 and exhibited 9.24- to 14.66-fold higher binding affinity than wildtype RBD. These data provide a reasonable explanation for the high frequencies of the three mutations in clinical sequencing data (Additional file 1: Fig. S5). Unlike N501Y, which induced tighter binding with ACE2 [51], S477N and L452R occurred at sites that were likely not in the ACE2 contact region (Fig. 5b). It is possible that the mutations altered the charge within the flexible loop region of the RBM, creating a more favourable environment for binding. Notably, the key antibody escape mutations K417N, N450K, E484K, E484Q, F490S, and R346S had limited effects on ACE2 binding affinity with fold changes between 0.4 and 2.5, suggesting that they were not accompanied by loss of fitness. Due to the stronger binding to ACE2 caused by the substitution N501Y and L452R, the binding affinity of B.1.351 triple mutant or B.1.617.1 double mutant RBD with ACE2 is nearly 3-fold higher than the wildtype RBD. Overall, our data is useful for understanding the evolutionary mechanism that governs the emergence of viral escape mutants.

### The key natural mutations were able to escape neutralization by COVID-19 convalescent donors

**Fig. 6:**
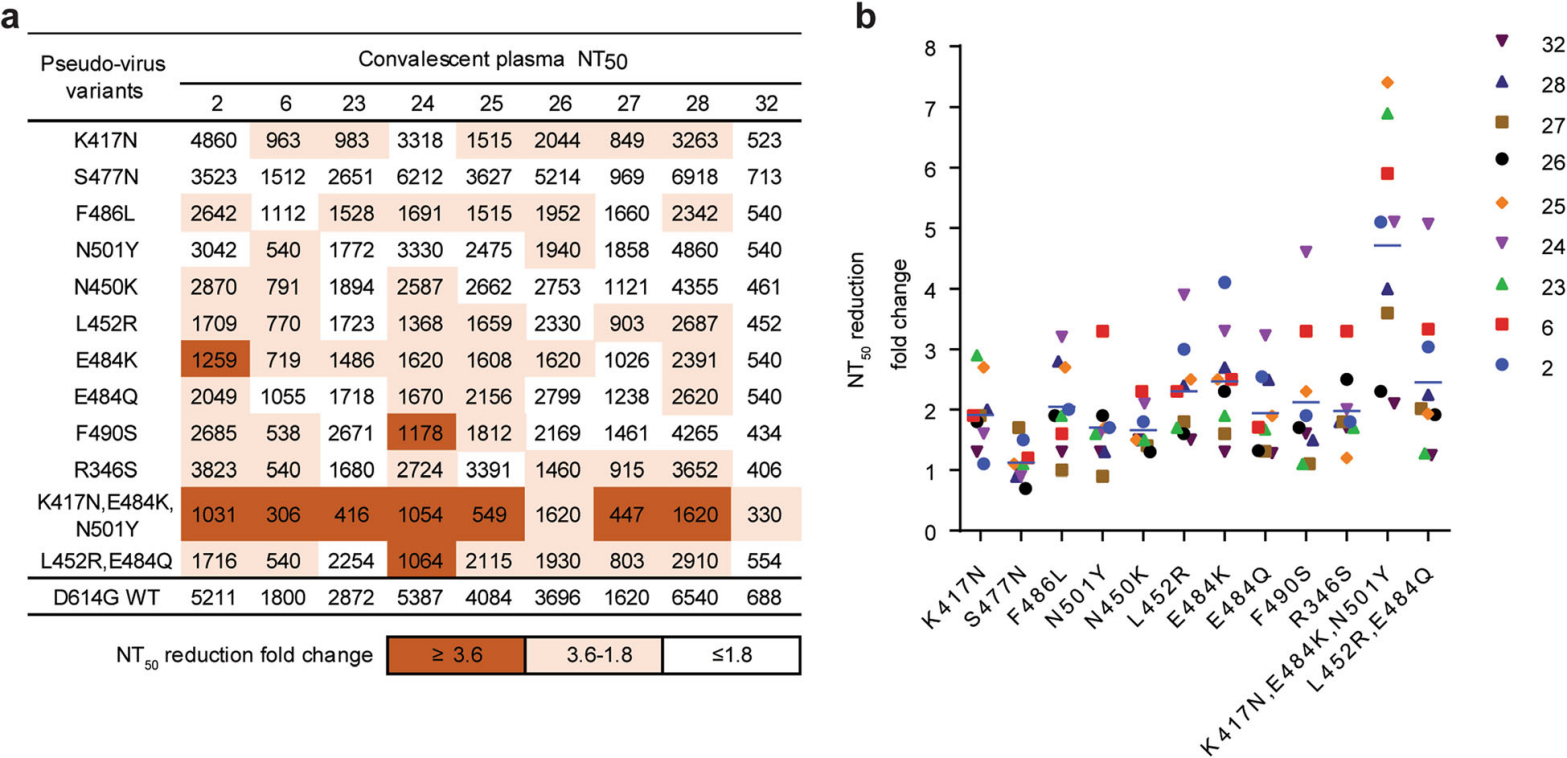
Effect of natural mutations on neutralizing activity of convalescent plasma. **a** Neutralization potency of nine convalescent plasma against pseudotyped variants. The data are presented as the highest plasma dilution giving a ≥ 50% inhibition of pseudotyped virus infection (*NT*_*50*_). *NT*_*50*_ values were calculated from three independent experiments. **b** The neutralizing antiserum titer against pseudotyped RBD variants decreases relative to wildtype pseudotyped virus. Neutralization fold change was calculated as follows: *NT*_*50*_ of the wildtype/*NT*_*50*_ of pseudotyped variants. The data represent one representative experiment of two independent experiments

To examine whether pseudoviruses with the key antigenic escape mutations conferred resistance to convalescent plasma from the first wave of SARS-CoV-2 infection in early 2020, nine out of 36 convalescent plasma samples (2, 6, 23, 24, 25, 26, 27, 28, and 32) were selected, and they exhibited different degrees of S-binding, receptor-blocking, and neutralizing activity (Additional file 1: Table. S8). The resistance profile of each human convalescent plasma sample was distinct, possibly because of the different repertoires of antigenic sites on the RBD targeted by polyclonal antibodies (Fig. 6a, b). Markedly, K417N, F486L, L452R, E484K, and R346S resulted in resistance to at least six plasma samples, as the NAb titers were approximately 2–4 times lower than those for the wildtype, indicating that NAbs targeting these key residues were enriched in human convalescent plasma. On the other hand, both F490S and N450K resulted in resistance to neutralization by plasma samples 6 and 24; in particular, the NAb titer of plasma sample 24 against F490S was reduced by 4.6 times. As expected, the single E484Q substitution had a milder impact than E484K*.* We observed that the substitution N501Y and S477N was neutralized in the same level as the wildtype by the majority of the plasma samples, in agreement with the previous studies [10, 52]. Finally, we demonstrated that compared with the wildtype residues, the N501Y, K417N, and E484K mutations in the B.1.351-variant pseudovirus dramatically reduced the neutralizing ability of all the plasma samples, with 2.1- to 7.4-fold reductions, which was also observed in previous studies [13, 53]. In addition, the combination of mutations resulted in more resistance than single mutations due to an additive effect caused by K417N and E484K. In contrast, the double L452R and E484Q mutations appeared a moderate decrease in neutralizing activity and failed to show an obvious additive effect compared to single mutation. One possible reason could be that the two key residues within antigenic 2 provide favorable interactions with the same cluster of antibodies, which were observed in our panel of mAbs (26-34L,24-12K, and 28-15L). Overall, these data suggest that circulating viruses with single mutations at antigen-binding hot spots could be resistant to neutralization by human convalescent plasma but that no single-amino acid mutation can enable robust escape. The evolution of co-mutations at distinct major antigenic sites is worthy of considerable attention.

## Discussion

New variants of SARS-CoV-2 continue to emerge; thus, it is critical to understand how the mutations within RBD facilitate the evading of immune protection or the acquiring of enhanced virulence. Here, functional mapping analysis revealed that the majority of RBD-specific NAbs target four antigenic sites (site 1, site 2, site 3, and site 4), which are consistent with the four structurally defined regions [4, 54, 55]. Molecular epidemiology analysis demonstrated that the immunodominant sites 1 and 2 are the most divergent regions, suggesting that variability in these sites is the main factor contributing to RBD antigenic evolution. In contrast, sites 3 and 4, which are in the core region, are more conserved and present a high genetic barrier to immune resistance, providing strong support for the development of antibodies and vaccine candidates that preserve these two conserved antigenic sites.

In this study, we functionally defined the key determinants that are critical for RBD-specific NAb recognition and further uncovered the numerous residues (often outside of the epitopes) essential for RBD-specific NAb recognition by affecting RBD folding or local antigenic conformation. These findings provide fundamental insights into antibody recognition of RBD, highlighting the dynamic and complex nature of RBD. Several binding hot spots that tend to be targeted by RBD-specific NAbs have been identified by alanine scanning: K417, F456, A475, F486, N487, N450, R452, E484, F490, and R346. Some of the hot spots have also been reported as the key binding determinants for NAbs by other groups [9, 56, 57]; thus, one application for our epitope maps is evaluating the possible immune evasion caused by RBD mutations which may be of immediate use. The valuable information that K417 are essential for multiple prototype VH3-53/3-66 NAb recognition allows us to re-interpret the potential significance of the mutation K417N and K417T present in B.1.351 and P.1. Differentiating with the previous research [56–58], our comprehensive escape mutation map is based on the natural substitutions of the binding hot spots identified in this study instead of mapping all mutations to the RBD, thus providing a promising means to rapidly track the key immune escape variants. Our comprehensive escape mutation map based on the natural substitutions of the binding hot spots not only confirms the widely circulating strains carrying important immune escape RBD mutations such as K417N, E484K, and L452R, but also facilitates the identification of new immune escape-enabling mutations that are already present at clinical isolates such as F486L, N450K, F490S, and R346S. For instance, as shown in Additional file 1: Fig. S7, the molecular epidemiology analysis as of June 2021 demonstrated that all the four mutations (F486L, N450K, F490S, and R346S) have occurred in the B.1.1.7 linage (Alpha) and F490S has appeared in other lineages of concern such as B.1.617.2 (Delta), B.1.429 (Epsilon), B.1.351 (Beta), and C.37 (Lambda). L452R and R346S co-mutations are present in some strains from different lineages in particular a new lineage C.36. These escape mutations or co-mutations may pose a new threat to current vaccine strategies in consideration of their little cost on receptor affinity (Fig. 5a). Given the in vitro neutralization data of RBD variants present in B.1.351 and B.1.617.1, our results suggest that SARS-CoV-2 variants carrying antigenic escape-enabling mutations at multiple residues may be resistant to vaccines or antibody-based therapeutics. Thus, special attention should be paid to the accumulation of co-mutations at distinct major antigenic sites during evolution.

Comparison of the hACE2 binding affinity between RBD mutants demonstrated that some antigenic escape-enabling mutations preserved or even enhanced hACE2 binding, which implies that these mutations are not accompanied by loss of fitness and are more likely to occur and spread quickly under immune pressure. The substitution L452R was particularly notable, as it also conferred resistance to multiple NAbs and human convalescent plasma. Therefore, circulating SARS-CoV-2 variants with L452R in the RBD region might be more infectious and less susceptible to NAbs and vaccines than variants without this mutation. Our previous study has also demonstrated that the SARS-CoV-2 RBD carrying L452K exhibits enhanced ACE2 binding. In contrast, the SARS-CoV RBD with K439 (452) L exhibits decreased ACE2 binding, indicating the involvement of a similar mechanism [23]. Co-mutations are needed to balance the fitness cost of antigenic change, as has been described for other viruses [59, 60]. Given the high mutation rate of this virus, additional combinations of mutations that are compatible with viral fitness or are associated with enhanced viral resistance are likely to arise [7]. Future work that combines investigations of antigenic mutations and receptor-binding affinity-affecting mutations should be performed to analyze antigenic changes, and research on viral fitness evolution is needed to monitor emerging high-risk strains.

## Conclusions

Our comprehensive antigenic maps of RBD targeting by a panel of representative NAbs provide valuable information for monitoring of the clinical consequences of SARS-CoV-2 evolution. Additionally, the reagents described here, particularly the three new broadly reactive NAbs, offer new options for antibody-based therapeutics and provide a useful set of tools for the evaluation of vaccines that are currently under investigation.

## Supplementary Information

**Additional file 1: Fig S1.** Characterization of antibody response induced by SARS-CoV-2 infection. **Fig S2.** Crossing binding and neutralizing activities of 28-26K, 25-F7 and 25-D9. **Fig S3.** Binding kinetics of RBD -specific mAbs with RBD from SARS-CoV-2 and SARS-CoV were measured by the Octet Red instrument. **Fig S4.** Common features of VH3-53/3-56 NAbs. **Fig S5.** Conservation of RBD residues. **Fig S6.** Binding kinetics of purified recombinant RBD mutants with ACE2-Fc were measured by BIAcore 8K. **Fig S7.** The molecular epidemiology analysis of four escape mutations F490S, N450K, R346S and F486L as of June 2021. **Table S3.** Antibody gene usage for selected NAbs. **Table S4.** hmAb panel percent binding to alanine mutants. **Table S6.** The alanine mutations resulted in less than 50% binding to the panel of conformation-dependent RBD-specific antibodies and ACE2 when analyzed by ELISA. **Table S7.** human mAb panel percent binding to natural RBD mutants. **Table S8.** Plasma samples from 9 individuals were screened by S-ECD and RBD binding titer and neutralizing titer against SARS-CoV-2 pseudovirus.

**Additional file 2: Table S1.** In vitro binding and neutralizing activity of RBD-specific mAbs.

**Additional file 3: Table S2.** Sequence analysis of RBD-specific mAbs.

**Additional file 4: Table S5.** The information of epitope targeted by RBD-specific mAbs.

## Data Availability

All data generated including all raw data are included in the main paper and its additional supporting files. The structures used for analysis and the amino acid sequences of the heavy chain and light chain of control mAbs were 6M0J (SARS-CoV-2 RBD/hACE2), 7C01 (SARS-CoV-2 RBD/CB6), 6WPT (SARS-CoV-2 S/S309), 7A5S (SARS-CoV-2 S/CR3022), and 7BZ5 (SARS-CoV-2 RBD/B38), which were downloaded from the PDB database (https://www.rcsb.org). The mutation information of the SARS-CoV-2 S protein was generated from CNCB-NGDC/the 2019nCoVR (https://ngdc.cncb.ac.cn/ncov/variation/spike) [29, 61]. All consensus full-length, nonredundant polyprotein sequences of clade 1, 2, and 3 sarbecoviruses, including human and animal isolates, are available from the GISAID database (https://www.gisaid.org) and the NCBI database.

## Abbreviations

COVID-19: Coronavirus disease 2019
SARS-CoV-2: Severe acute respiratory syndrome coronavirus-2
SARS-CoV: Severe acute respiratory syndrome coronavirus
ACE2: Angiotensin-converting enzyme 2
ELISA: Enzyme-linked immunosorbent assay
RBD: Receptor-binding domain
NAbs: Neutralizing antibodies
RBM: Receptor-binding motif
KD: Equilibrium dissociation constant
GISAID: Global Initiative on Sharing All Influenza Data
NCBI: National Center for Biotechnology Information
NGDC: National Genomics Data Center
VH: Heavy-chain variable domain
VL: Light-chain variable domain
