# Supplementary material for: Comprehensive mapping of binding hot spots of SARS-CoV-2 RBD-specific neutralizing antibodies for tracking immune escape variants
Source: Genome Med. 2021 Oct 14;13:164. doi: 10.1186/s13073-021-00985-w (PMC8515915; doi:10.1186/s13073-021-00985-w)
Supplement: Supplementary file 1 — Additional file 1: Fig S1. Characterization of antibody response induced by SARS-CoV-2 infection. Fig S2. Crossing binding and neutralizing activities of 28-26K, 25-F7 and 25-D9. Fig S3. Binding kinetics of RBD -specific mAbs with RBD from SARS-CoV-2 and SARS-CoV were measured by the Octet Red instrument. Fig S4. Common features of VH3-53/3-56 NAbs. Fig S5. Conservation of RBD residues. Fig S6. Binding kinetics of purified recombinant RBD mutants with ACE2-Fc were measured by BIAcore 8K. Fig S7. The molecular epidemiology analysis of four escape mutations F490S, N450K, R346S and F486L as of June 2021. Table S3. Antibody gene usage for selected NAbs. Table S4. hmAb panel percent binding to alanine mutants. Table S6. The alanine mutations resulted in less than 50% binding to the panel of conformation-dependent RBD-specific antibodies and ACE2 when analyzed by ELISA. Table S7. human mAb panel percent binding to natural RBD mutants. Table S8. Plasma samples from 9 individuals were screened by S-ECD and RBD binding titer and neutralizing titer against SARS-CoV-2 pseudovirus. [file 13073_2021_985_MOESM1_ESM.docx]

**Additional file 1**


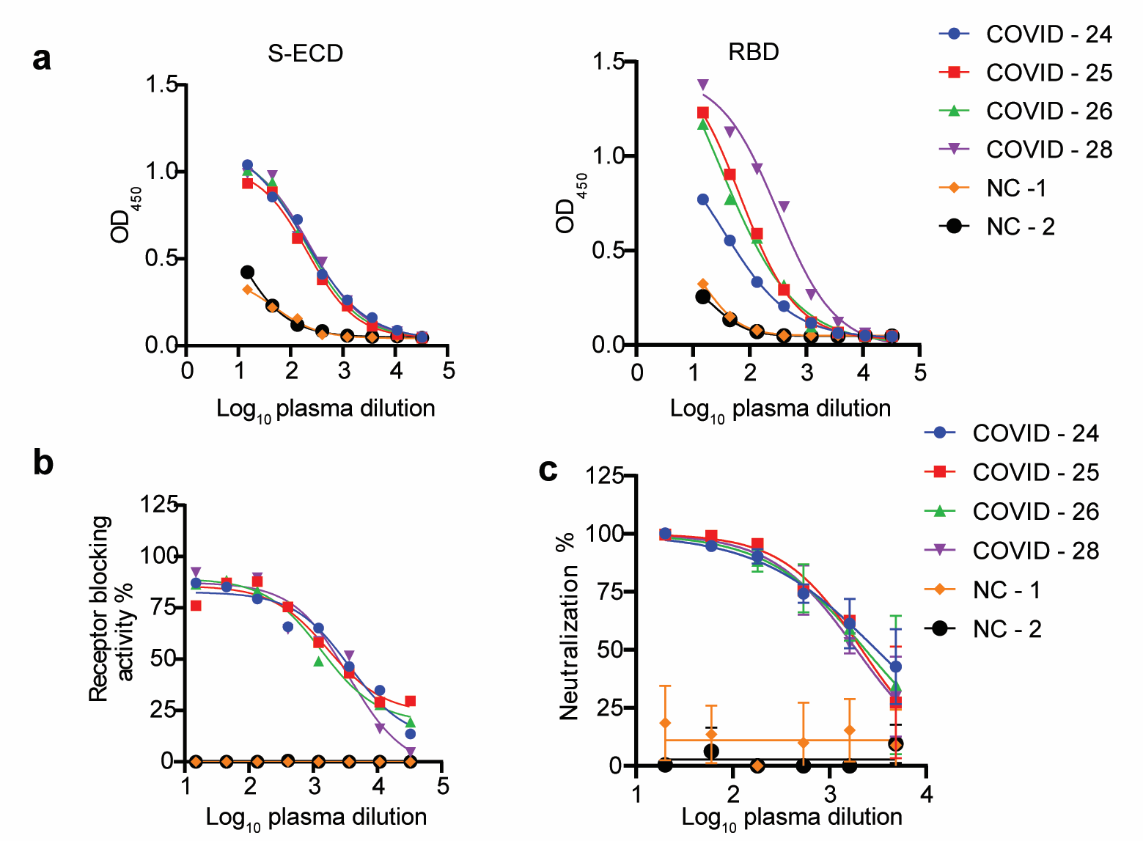


**Fig S1:** Characterization of antibody response induced by SARS-CoV-2 infection. **a** The ELSIA binding curves for four COVID-19 convalescent patients (24, 25, 26 and 28) and two healthy people. Antibody titers was assessed against S-ECD (extracellular domain of S protein) proteins and RBD region of SARS-CoV-2. **b** The effect of convalescent plasma on the binding of RBD to ACE2. **c** The neutralization curves for four COVID-19 convalescent patients (24,25,26 and 28) and two healthy people against SARS-CoVD-2 pseudovirus. The data represent one representative experiment of two independent experiments.


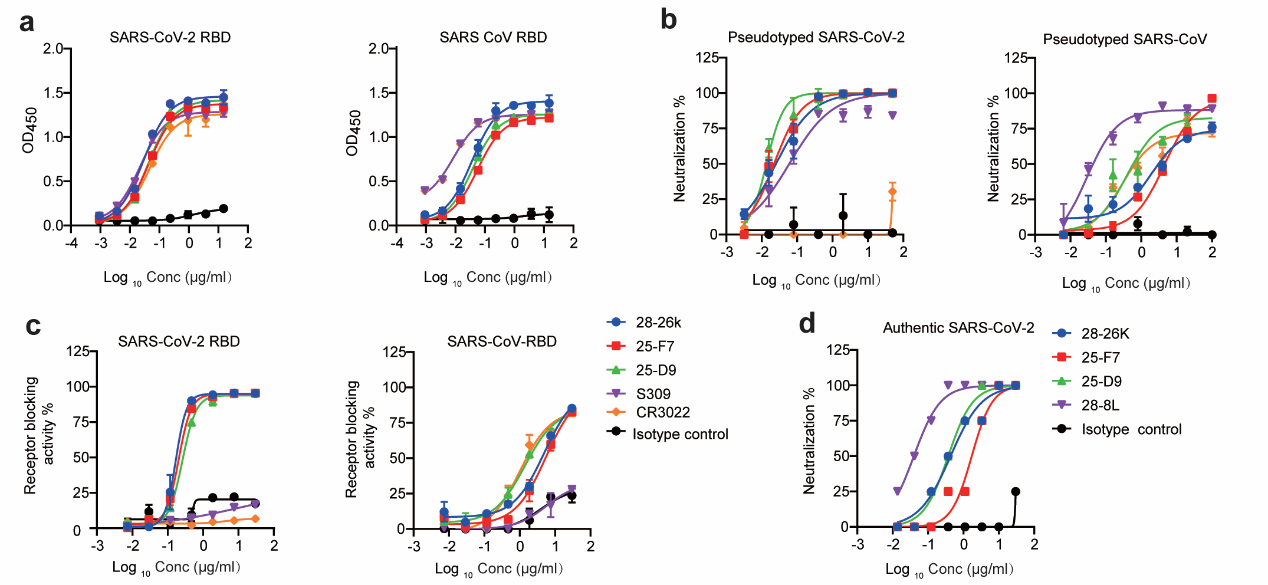


**Fig S2:** Crossing binding and neutralizing activities of 28-26K, 25-F7 and 25-D9. **a** The binding curves of 28-26K, 25-F7 and 25-D9 to SARS-CoV-2 RBD (left) and SARS-CoV RBD (right) are shown. The data was shown as mean±SD. **b** The neutralization curves for 28-26K, 25-F7 and 25-D9 against SARS-CoV-2 pseudo-virus (left) and SARS-CoV pseudo-virus (right). **c** ACE2 receptor blocking activity of 28-26K, 25-F7 and 25-D9. Left, SARS-CoV-2 RBD; right, SARS-CoV RBD. **d** The neutralizing activity of 28-26K, 25-F7 and 25-D9 against SARS-CoV-2 authentic virus. Three previously described S309, CR3022, and 28-8L were used as control. For **a-d**, values shown are the means of triplicate. The curves were fit by nonlinear regression in Graphpad Prism.


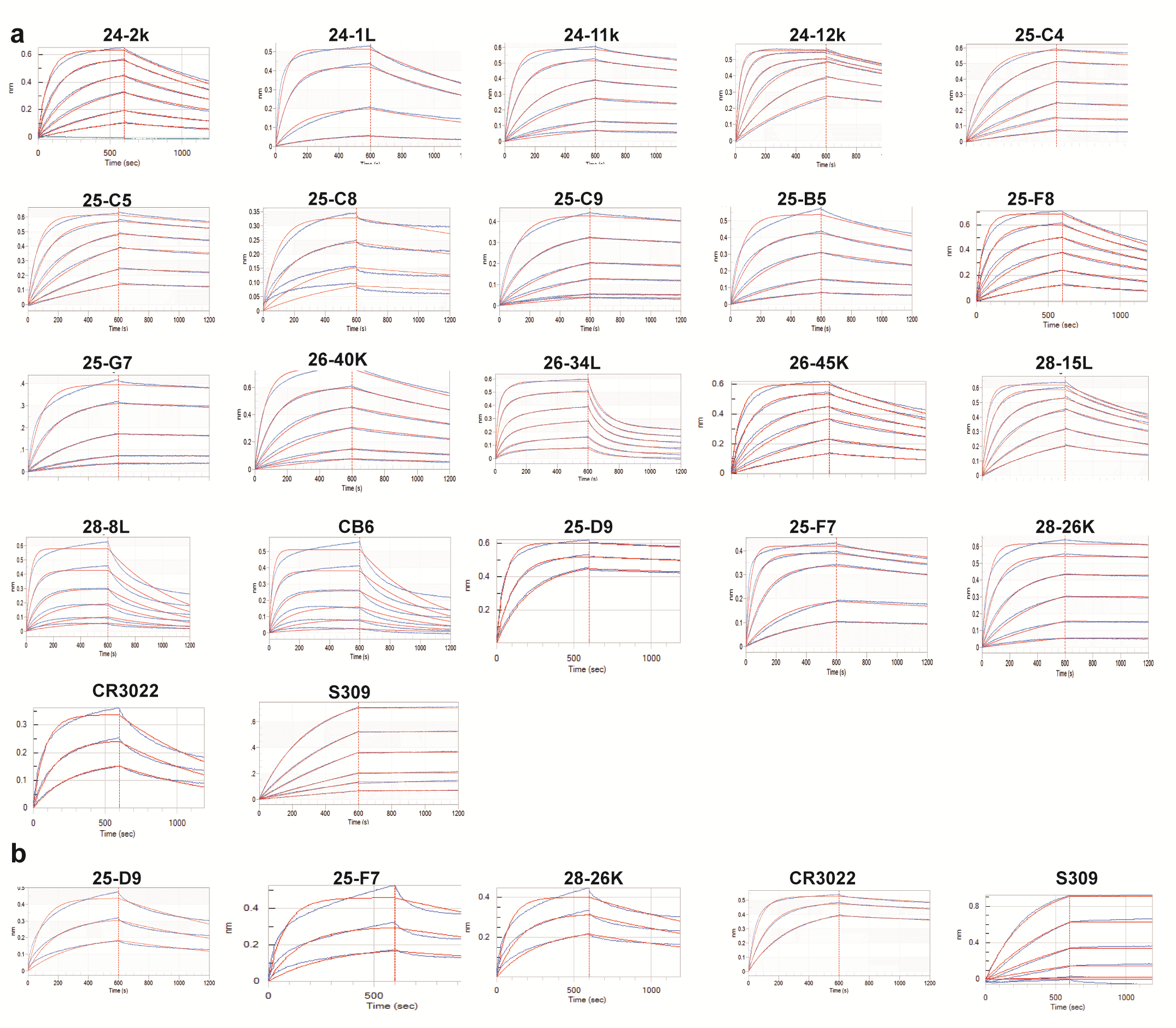


**Fig S3:** Binding kinetics of RBD -specific mAbs with RBD from SARS-CoV-2 and SARS-CoV were measured by the Octet Red instrument. **a** Binding kinetics of purified recombinant mAbs with SARS-CoV-2 RBD. **b** Binding kinetics of cross-reactivity mAbs with SARS-CoV RBD. Blue curves are the experimental trace, and red curves are the best global fits to the data used to calculate the KD. The lines represent 3.125 nM, 6.25 nM, 12.5 nM, 25 nM, 50 nM and 100 nM concentrations of RBD. The data were double reference subtracted and fit using a 1:1 binding model.


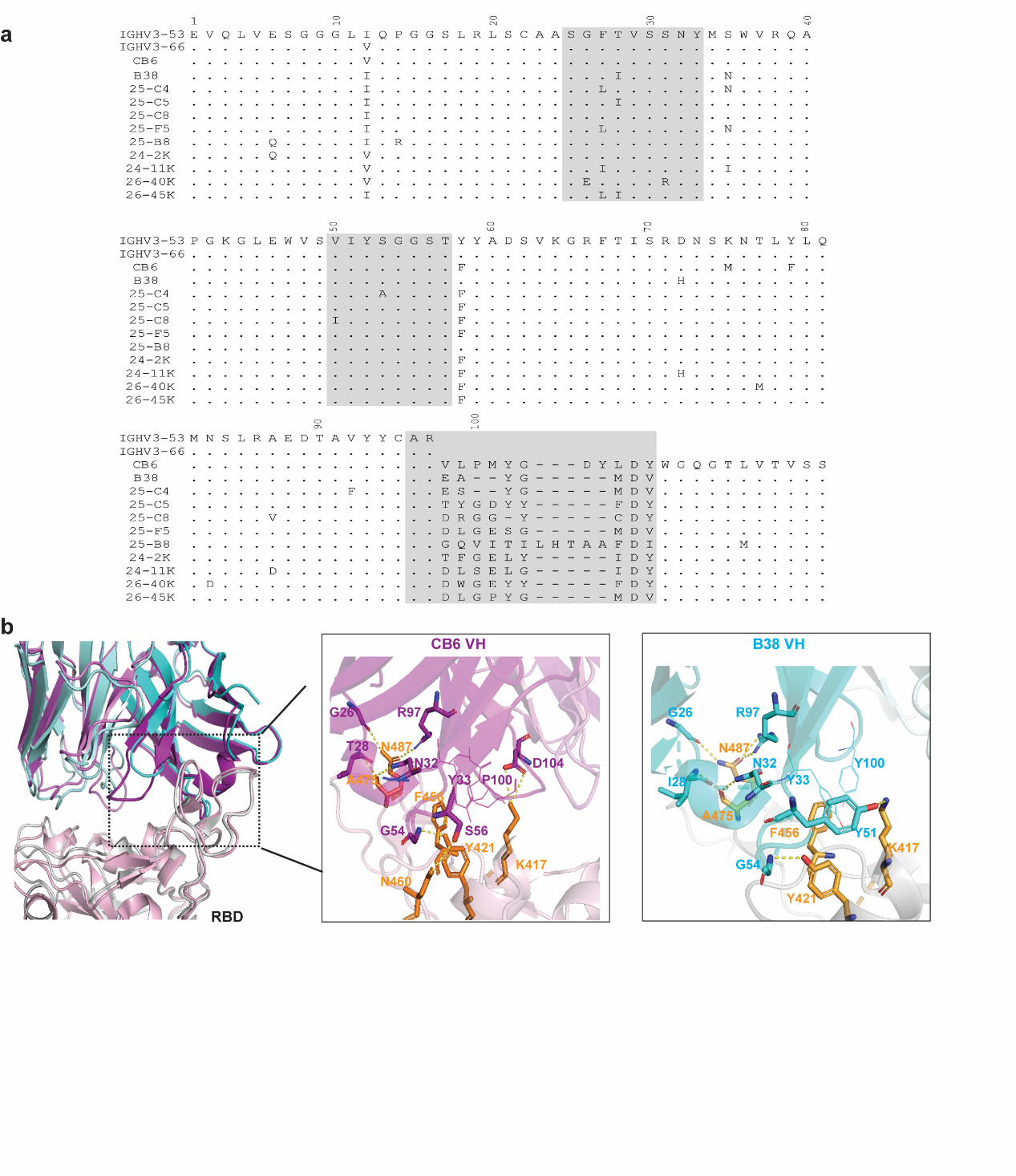


**Fig S4:** Common features of VH3-53/3-56 NAbs. **a** Alignment of the VH amino acid sequences of 3-53/3-56 with its germline configuration. The CDR regions are highlighted in grey. Dots indicate identical residues; Somatic mutations are shown. **b** Structural alignment of CB6 and B38 binding with RBD. CB6-RBD complex (PDB ID: 7C01) is superimposed on B38- RBD (PDB ID: 7BZ5). CB6-RBD complex: magenta, CB6 VH; violet, CB6 VL; Light pink, RBD. B38-RBD complex: cyan, B38 VH; pale cyan; RBD, grey. Key residues on RBD highlighted in orange sticks. Polar interactions are indicated by yellow dashed lines.


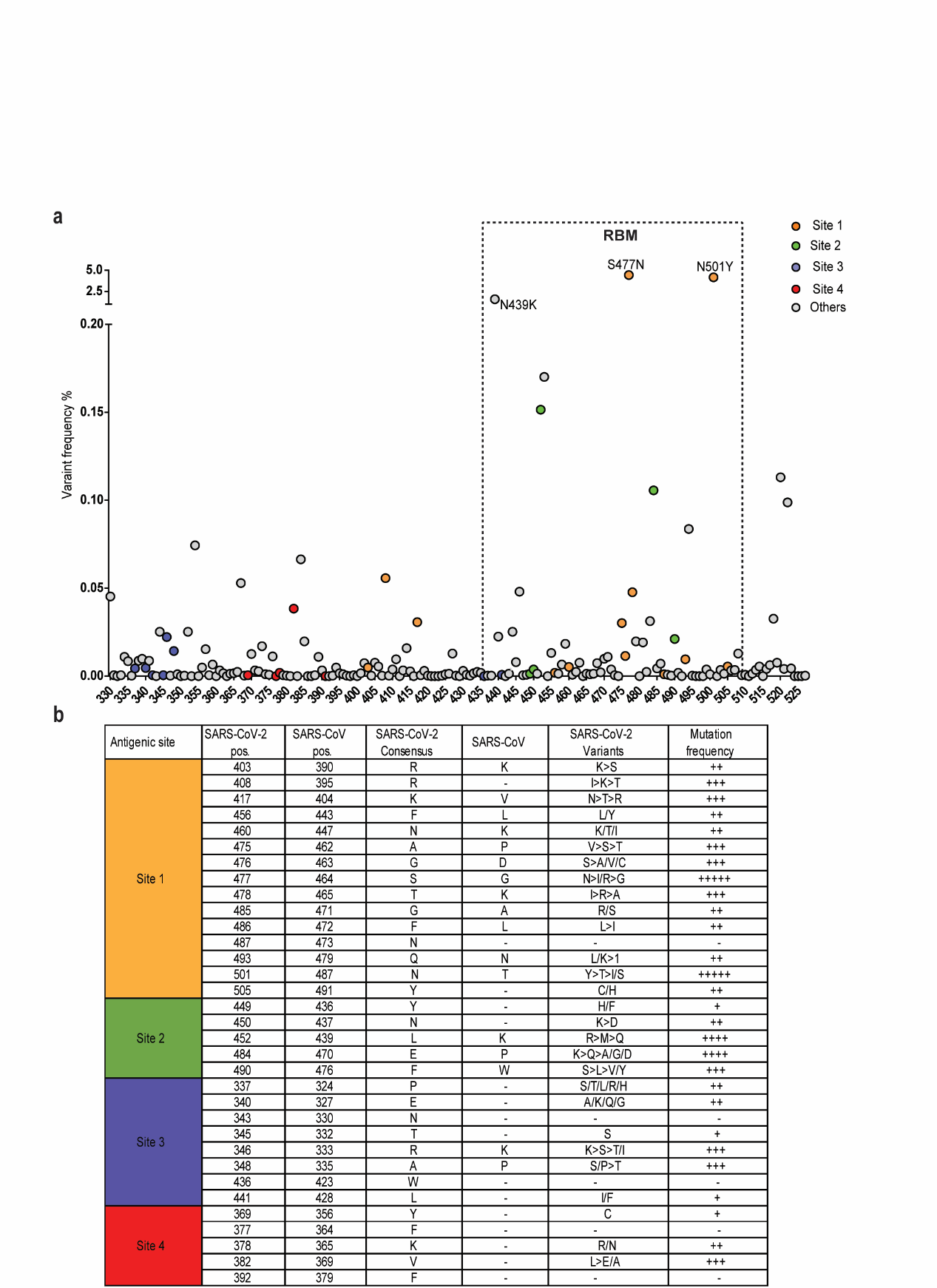


**Fig S5:** Conservation of RBD residues. SARS-CoV-2 genome sequences (n=364,409) retrieved from GISAID and Genbank on January 19^th^ 2021 (n=11,839) were used to annotate variants of the spike glycoprotein. 3272 mutations had been identified in the S gene of SARS-CoV-2 isolated from humans (CNCB-NGDC, 2021; GISAID, 2021). These mutations lead to 1961 amino acid changes, including 259 substitutions in the RBD region. **a** Variants are plotted by amino acid position. Each dot is a distinct variant. Color encodes four antigenic sites. Variants are labeled if their prevalence is greater than 1%. **b** Variability at neutralizing antibody binding residues. “+++++” means > 1%; “++++” means 0.1-1%; “+++” 0.01-0.1%; “++” 0.001-0.01; “+” means 0.0001-0.001; “-” means no varation. “>”, more; “/”, comparable.


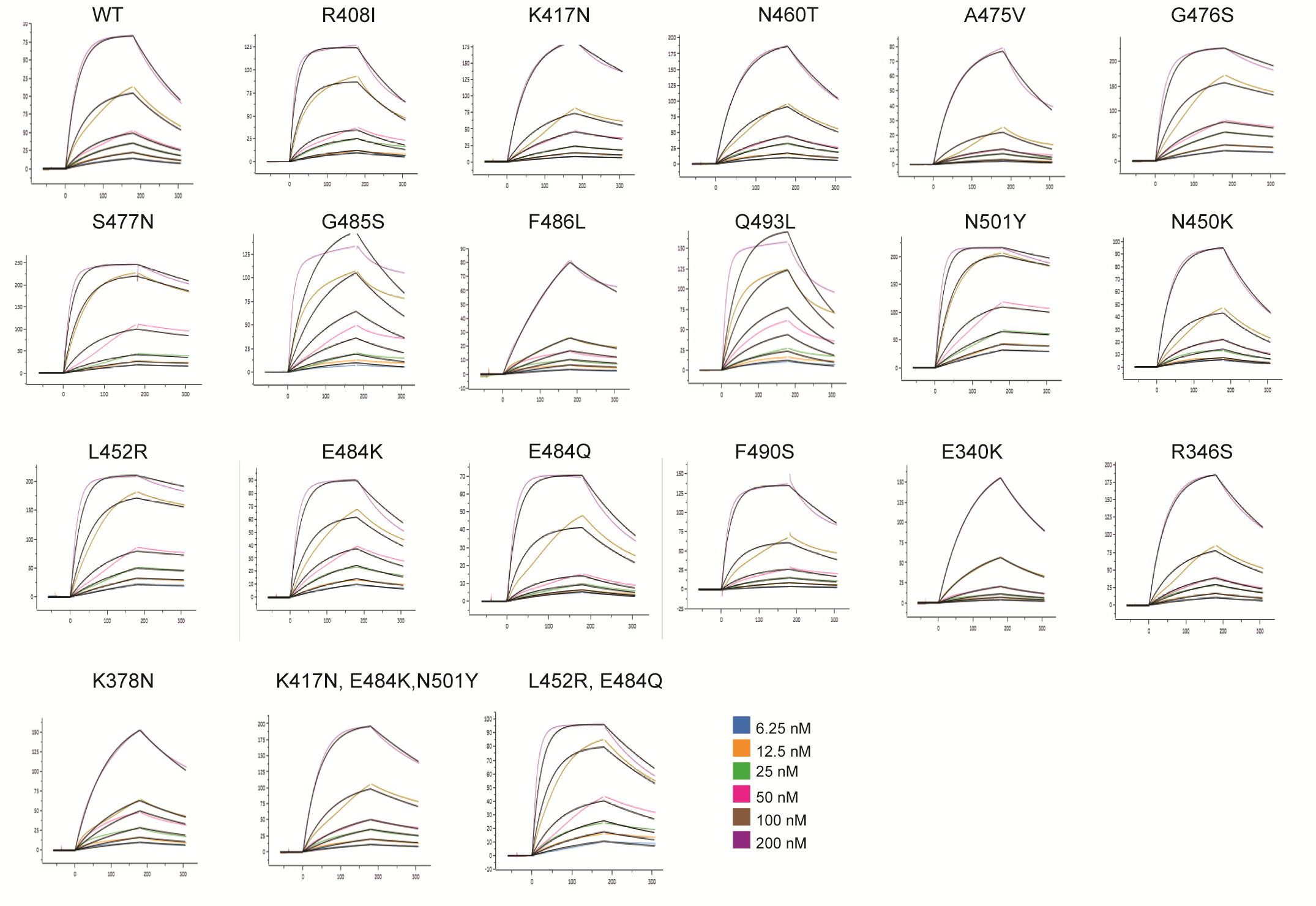


**Fig S6:** Binding kinetics of purified recombinant RBD mutants with ACE2-FC were measured by BIAcore 8K. The lines represent 6.25 nM, 12.5 nM, 25 nM, 50 nM, 100 nM, and 200 nM nM concentrations of RBD mutants and wildtype. The data were double reference subtracted and fit using a 1:1 binding model. WT, wildtype RBD.


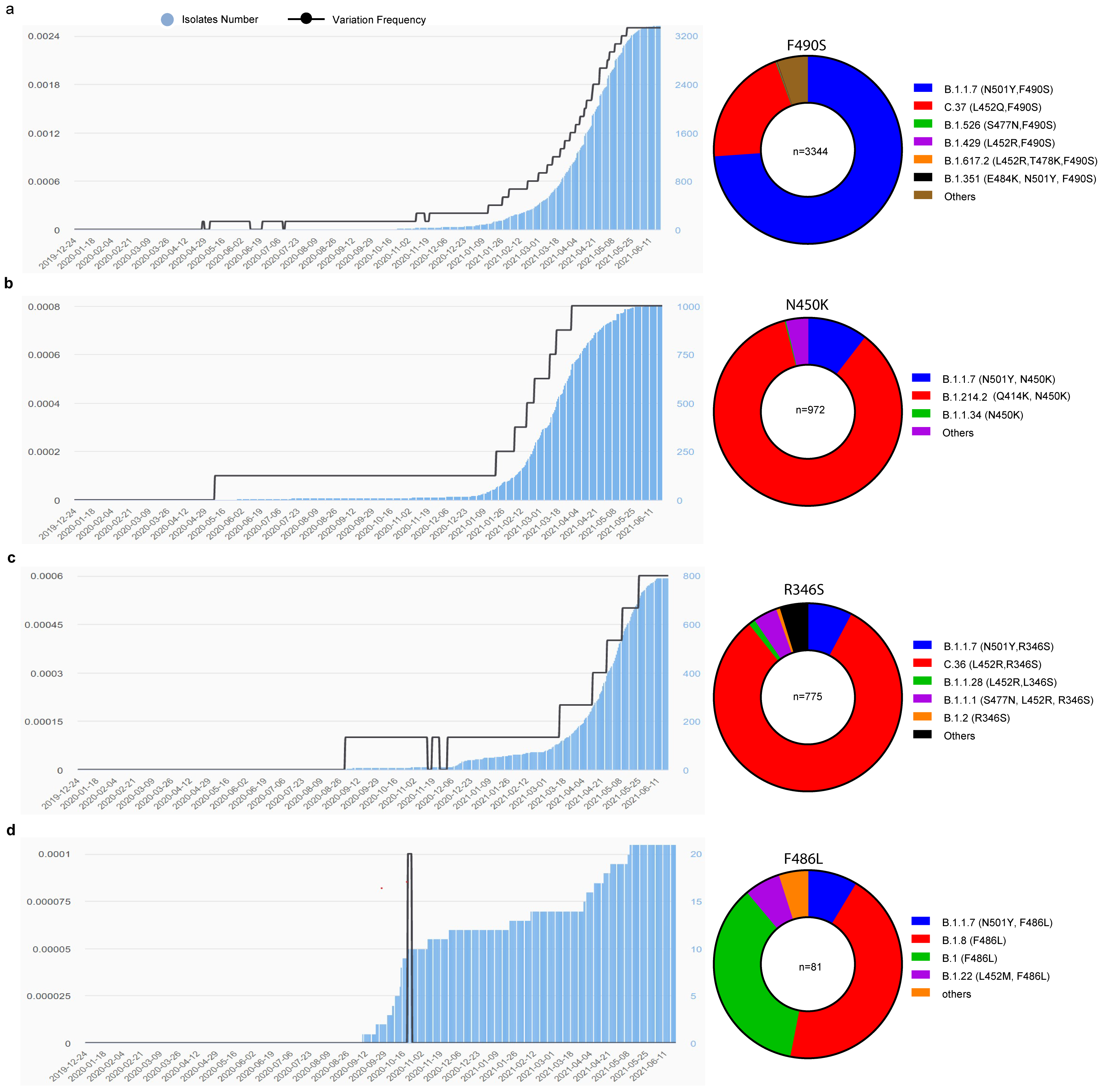


**Fig S7:** The molecular epidemiology analysis of four escape mutations F490S, N450K, R346S and F486L as of June 2021. **a-d** Time frequency and lineage analysis of the variations at F490, N450, R346 and F486. Left, time frequency analysis; right, the percentage of mutants in different lineages. The time frequency data were generated from The National Genomics Data Center (NGDC). The RBD mutations present in evaluated SARS-CoV-2 genomic sequences deposited in GISAID and GenBank as of June 18, 2021 were analyzed by CoVsurver.

**Table S3:** Antibody gene usage for selected NAbs.

mAbs are grouped based on competitive binding experiments. The lineage analysis and the percent identity for each predicted gene usage based on IMGT/VQUEST predictions. VH, heavy chain variable region gene; JH, heavy chain joining region gene; VL, light chain variable region gene; JL, light chain joining region gene.

**Table S4: hmAb panel percent binding to alanine mutants.**

****Coloring corresponds to the reactivity to each RBD mutant relative to WT with ≤ 30% dark orange, 30±70% light salmonand and ≥ 70% white.

**Table S6:** The alanine mutations resulted in less than 50% binding to the panel of conformation-dependent RBD-specific antibodies and ACE2 when analyzed by ELISA.

Coloring corresponds to the reactivity to each RBD mutant with < 30% dark orange, 30±70% light salmonand > 70% white. Residues located in the core region are highlighted in green; in the RBM region are highlighted in cyan. Anti- His tag was used to verify the expression level.

**Table S7: human mAb panel percent binding to natural RBD mutants.**

Coloring corresponds to the reactivity to each RBD mutant relative to WT with ≤ 30% dark orange, 30±70% light salmonand and ≥ 70% white.

**Table S8:** Plasma samples from 9 individuals were screened by S-ECD and RBD binding titer and neutralizing titer against SARS-CoV-2 pseudovirus.

| **Plasma** | **Ab titer** | | **BT_50_** | **NT_50_** |
| --- | --- | --- | --- | --- |
|  | **S-ECD** | **RBD** |  |  |
| 2 | 3645 | 1215 | 5854 | 7118 |
| 6 | 1215 | 405 | 874 | 1758 |
| 23 | 3645 | 1215 | 1010 | 2521 |
| 24 | 3645 | 1215 | 3661 | 6128 |
| 25 | 3645 | 1215 | 2138 | 4326 |
| 26 | 3645 | 1215 | 1335 | 4723 |
| 27 | 1215 | 405 | 373 | 1957 |
| 28 | 3645 | 3645 | 2812 | 4455 |
| 32 | 405 | 135 | 45 | 540 |

BT_50_, the highest**plasma dilution** giving a ≥ 50% inhibition of receptor blocking activity. NT_50_, the highest**plasma dilution** giving a ≥ 50% inhibition of pseudovirus virus infection.
